# Enhanced Desulfurization Performance of ZIF−8/PEG MMMs: Effect of ZIF−8 Particle Size

**DOI:** 10.3390/membranes13050515

**Published:** 2023-05-15

**Authors:** Xia Zhan, Kaixiang Gao, Yucheng Jia, Wen Deng, Ning Liu, Xuebin Guo, Hehe Li, Jiding Li

**Affiliations:** 1 China Food Flavor and Nutrition Health Innovation Center, Beijing Technology and Business University, Beijing 100048, China; 2 Key Laboratory of Cleaner Production and Integrated Resource Utilization of China National Light Industry, Beijing Technology and Business University, Beijing 100048, China; 3 Department of Chemical Engineering, Tsinghua University, Beijing 100084, China

**Keywords:** ZIF−8, particle size, pervaporation, desulfurization, transport mechanism

## Abstract

Constructing efficient and continuous transport pathways in membranes is a promising and challenging way to achieve the desired performance in the pervaporation process. The incorporation of various metal–organic frameworks (MOFs) into polymer membranes provided selective and fast transport channels and enhanced the separation performance of polymeric membranes. Particle size and surface properties are strongly related to the random distribution and possible agglomeration of MOFs particles, which may lead to poor connectivity between adjacent MOFs-based nanoparticles and result in low-efficiency molecular transport in the membrane. In this work, ZIF−8 particles with different particle sizes were physically filled into PEG to fabricate mixed matrix membranes (MMMs) for desulfurization via pervaporation. The micro-structures and physi-/chemical properties of different ZIF−8 particles, along with their corresponding MMMs, were systematically characterized by SEM, FT-IR, XRD, BET, etc. It was found that ZIF−8 with different particle sizes showed similar crystalline structures and surface areas, while larger ZIF−8 particles possessed more micro-pores and fewer meso-/macro-pores than did the smaller particles. ZIF−8 showed preferential adsorption for thiophene rather than n−heptane molecules, and the diffusion coefficient of thiophene was larger than that of thiophene in ZIF−8, based on molecular simulation. PEG MMMs with larger ZIF−8 particles showed a higher sulfur enrichment factor, but a lower permeation flux than that found with smaller particles. This might be ascribed to the fact that larger ZIF−8 particles provided more and longer selective transport channels in one single particle. Moreover, the number of ZIF−8−L particles in MMMs was smaller than the number of smaller ones with the same particle loading, which might weaken the connectivity between adjacent ZIF−8−L nanoparticles and result in low-efficiency molecular transport in the membrane. Moreover, the surface area available for mass transport was smaller for MMMs with ZIF−8−L particles due to the smaller specific surface area of the ZIF−8−L particles, which might also result in lower permeability in ZIF−8−L/PEG MMMs. The ZIF−8−L/PEG MMMs exhibited enhanced pervaporation performance, with a sulfur enrichment factor of 22.5 and a permeation flux of 183.2 g/(m^−2^·h^−1^), increasing by 57% and 389% compared with the results for pure PEG membrane, respectively. The effects of ZIF−8 loading, feed temperature, and concentration on desulfurization performance were also studied. This work might provide some new insights into the effect of particle size on desulfurization performance and the transport mechanism in MMMs.

## 1. Introduction

Sulfur compounds in gasoline will lead to severe environmental problems, such as acid rain, air pollution, etc. Therefore, strict restrictions on the sulfur content in gasoline have been imposed by many governments, and the R&D of desulfurization technology has captured worldwide attention. Compared with traditional catalytic hydrodesulfurization technology, pervaporation exhibits distinct advantages, such as low octane loss and energy consumption, flexible and environmentally friendly operation, etc., [1].

The membrane is the key factor determining the final separation performance in the pervaporation process. High-performance desulfurization membranes with fast and selective sorption and diffusion of sulfur-containing molecules are crucial in pervaporation process according to solution-diffusion mechanism. Moreover, incorporating porous nanoparticles into polymer membranes to prepare mixed matrix membranes (MMMs) has proved to be an efficient strategy to improve separation performance. MMMs exhibited outstanding pervaporation performance compared with pristine polymeric membranes and showed promising applications in a variety of separation fields [2,3,4,5,6,7,8], including azeotropic separation, the dehydration of organic solvents, the selective separation of organics from aqueous solution, etc. Metal-organic frameworks (MOFs), which are composed of organic ligands and metal ions or metal oxide clusters based on coordination bonds, is an attractive class of porous materials due to their high porosity, large surface areas, and tunable physi-/chemical properties [9,10,11,12,13,14,15,16,17,18,19,20]. Moreover, MOFs exhibit good compatibility with a polymer matrix due to their intrinsic inorganic–organic hybrid properties, which could be widely applied to prepare MMMs. Various MOFs, such as ZIF−8 [21], MOF-505 [10], MOF-508a [22], CPO-27-Ni [23], MIL-101(Cr) [24], CuBTC [25], UiO-67-bpydc [26], etc., have been employed as inorganic fillers of polymeric membranes for desulfurization, gas separation [6], water treatment [7], and so on. The enhanced desulfurization performance achieved by incorporating MOFs into polymer membranes was usually ascribed to the improvement of sorption selectivity and/or facilitating transport channels between adjacent MOFs particles. Most studies have focused on the effect of MOFs types and loading on desulfurization performance, but few have concentrated on the effect of particle size on transport channel connectivity and desulfurization performance. Particle size and surface properties are strongly related to the random distribution and possible agglomeration of MOFs particles [27,28,29], which may lead to poor connectivity between adjacent MOFs-based nanoparticles and result in low-efficiency mass transport in the membrane. Therefore, it is meaningful to investigate the effect of particle size on desulfurization performance and to promote the connectivity of transport channels provided by MOFs particles to achieve high separation performance.

In this work, ZIF−8 particles with three different particle sizes were employed to fabricate ZIF−8/PEG MMMs for gasoline desulfurization via pervaporation. The micro-structures and physi-/chemical properties of ZIF−8 with different particle size, along with their corresponding MMMs, were systematically characterized. The sorption and diffusion behavior of the thiophene/n−heptane in the ZIF−8 particles was investigated based on molecular simulation. The influence of ZIF−8 particle size, particle loading, feed temperature, and concentration on the desulfurization performance was investigated in detail.

## 2. Materials and Methods

### 2.1. Materials

Zinc nitrate hexahydrate (Zn(NO_3_)_2_·6H_2_O, 99%) was purchased from Tianjin FuChen Chemical Reagent Co., Ltd., Tianjin, China, and 2-methylimidazole (Hmim, C_4_H_6_N_2_, 98%) was obtained from TCI Development Co., Ltd., Tokyo, Japan. Polyethylene glycol (PEG, Mw = 200,000 g/mol) was obtained from Sigma-Aldrich Trading Co., Ltd., Shanghai, China. Methanol (MeOH, AR) was purchased from Xilong Chemical Co., Ltd., Tianjin, China. Citric acid was obtained from Sinopharm Chemical Reagent Co., Ltd. (Shanghai, China). Trimethylamine (TMA) aqueous solution was purchased from Yongda Chemical Reagent Co., Ltd. (Tianjin, China). n−heptane (AR) was obtained from Sinopharm Chemical Reagent Co., Ltd., Shanghai, China. Thiophene (AR) was purchased from MackLin Biochemistry Technology Co., Ltd., Shanghai, China. The PVDF porous support membrane was self-made in our own laboratory.

### 2.2. Membrane Preparation

#### 2.2.1. Synthesis of ZIF−8 Particles

ZIF−8 nanoparticles were prepared according to the preparation process reported in the literature [30]. The zinc nitrate hexahydrate (2.5 mmol) and a specific amount of 2-methylimidazole was dissolved in 50 mL methanol (denoted as A and B, respectively). Then, A was added dropwise into B. Next, the mixture solution was vigorously stirred for 1.5 h. The turbid solution was centrifuged and washed with methanol three times. The product was dried at room temperature in a vacuum oven for 12 h. The dried particles resulted in ZIF−8 particles. The molar ratio of Zn^2+^ to Hmim was controlled to 2:1, 3:1, and 4:1, and the obtained particles were denoted as ZIF−8−L, ZIF−8−M, and ZIF−8−S, respectively. 

#### 2.2.2. Membrane Preparation

The membrane preparation process is shown in Figure S1. First, polyethylene glycol (PEG) was dissolved in deionized water under vigorous stirring for 2 h at 70 °C. Then, different amounts of ZIF−8 were dispersed into the PEG solution, and the mixed solution was sonicated for 20 min to alleviate particle aggregation and sedimentation. The resulting homogeneous solution was stirred for an additional 4 h and kept at 70 °C. The solution was degassed under vacuum after mixing for approximately three minutes. Finally, a glass rod was used to rapidly cast the solution onto a porous PVDF substrate membrane adhered on glass plates. The membranes were then dried overnight at room temperature and crosslinked at 80 °C for 5 h in a vacuum oven to remove any residual solvent. To improve the dispersion of ZIF−8 in the PEG matrix, pre-treatment with grinding of ZIF−8 particles and pre-crosslinking of PEG were employed.

### 2.3. Material Characterization

The BET surface area, pore volume, and pore size distribution were calculated based on the nitrogen adsorption isotherms at 77 K using an ASAP2020 (Micromeritics, Atlanta, GA, USA) gas sorption instrument. The morphology of the ZIF−8 particles and various membranes was investigated using a JSM-7410F Field Emission Microscope (JEOL, Tokyo, Japan) equipped with EDS mapping analysis. The FT-IR was used to study the chemical structure of ZIF−8 and the membranes using a Nicolet iS10 FT-IR Spectrometer (Thermo Fisher Scientific, Waltham, MA, USA) in the scanning range of 4000 and 400 cm^−1^. The crystalline properties of ZIF−8 and the membranes were investigated by XRD with a D8 ADVANCE X-ray diffractometer (Bruker, Karlsruhe, Germany) in the range of 5 to 50° at a rate of 6°/min.

Thermal gravimetric analysis (TGA) was used to evaluate the thermal stability of ZIF−8, PEG, and ZIF−8/PEG MMMs using a STA 409C/3/F Simultaneous Thermal Analyzer (Netzsch Corporation, Free State of Bavaria, Germany) in the range of room temperature to 800 °C at a heating rate of 20 °C/min with a nitrogen flow. Differential scanning calorimetry (DSC) measurements were conducted using a DSC Q20 (TA Instruments, New Castle, DE, USA) under nitrogen atmosphere from −80 °C to 100 °C. Both cooling and heating temperature scans were operated at the rate of 10 °C/min under a dry nitrogen atmosphere. The swelling degree of the membranes was calculated as follows. The membranes were immersed in thiophene/n−heptane solution (500 ppm) at room temperature for 48 h, the surface of the samples was quickly wiped with tissue paper to remove the excess liquid, and the samples were weighed as quickly as possible. The samples were then dried and weighed. The swelling degree (SD) was calculated using the following equation:

$$SD=\frac{M-M_{0}}{M_{0}}\times 100\%$$

where *M* and *M*_0_ are the masses of the swollen and dry membranes, respectively.

### 2.4. Pervaporation Performance

The pervaporation apparatus was self-made in our own lab, as shown in Figure S2. The separation performance was evaluated with two indexes: permeate flux (J) and enrichment factor (*β*). Thiophene/n−heptane solution was used as the feed mixture, which was kept at temperatures in the range of 60–80 °C. The permeate flux was obtained by weighing the permeate collected in a cold trap for a given amount of time. The concentrations of the permeates and the feed were determined using GC7900 chromatography. The enrichment factor and permeation flux were calculated according to the following equations [22].

$$\beta =\frac{\omega^{P}}{\omega^{F}}$$

where *β* represented the enrichment factor. It is the ratio of weight percents of the thiophene in the permeation and feed sides.

$$J=\frac{Q}{T\cdot S}$$

where *J* represented the permeate flux, *Q* represented the weight of the liquid collected in the cold traps, *S* represented the effective membrane area, and *T* represented the experimental time.

The apparent activation energy was determined based on the relationship between temperature and permeation flux, according to Arrhenius equation:

$$J_{i}=J_{oi}\exp\left(\frac{-E_{p}}{RT}\right)$$

where *J_oi_* represented the pre-exponential factor; and *E_p_* is the apparent activation energy of flux, which can be calculated from the slope of the fitted line.

### 2.5. Packing Models and Simulation Details

The simulation was conducted using Materials Studio software. The lattice information of ZIF−8 was obtained from the Cambridge Crystallographic Data Center (CCDC). The size of the ZIF−8 cell was 33.982 × 33.982 × 33.982 Å with a 2 × 2 × 2 unit cell. The adsorption isotherm diagrams of thiophene and n−heptane in ZIF−8 were simulated using the grand canonical Monte Carlo (GCMC) method at 333 K. 20 different pressures were set for each adsorption process, and one million calculation steps using GCMC were performed with one million steps for the primary equilibration period. 

The diffusion behaviors of thiophene and n−heptane in ZIF−8 were investigated based on the molecular dynamic simulation in the Forcite module. Five thiophene or n−heptane molecules were inserted into the ZIF−8 cell. First, 5000 iterations of the geometric optimization process were performed to optimize the geometric structure of the model. Then, 200 ps NPT (fixed atom number, system pressure, and temperature) at 0.0001 GPa, with a time step of 1 fs, was performed to bring the system close to its true density. Another 1000 ps NVT (fixed atom number, system volume, and temperature) with a time step of 1 fs, was conducted to investigate the molecular dynamic properties of the thiophene/n−heptane molecules. The charges of ZIF−8 were applied using the QEq method. The universal force field and the current charge were used to perform all the model calculations [31,32,33]. The cutoff was defined as 12.5 Å. 

## 3. Results and Discussion

### 3.1. Characterization of ZIF−8/PEG MMMs

#### 3.1.1. ZIF−8 Particles

**Figure 1:**
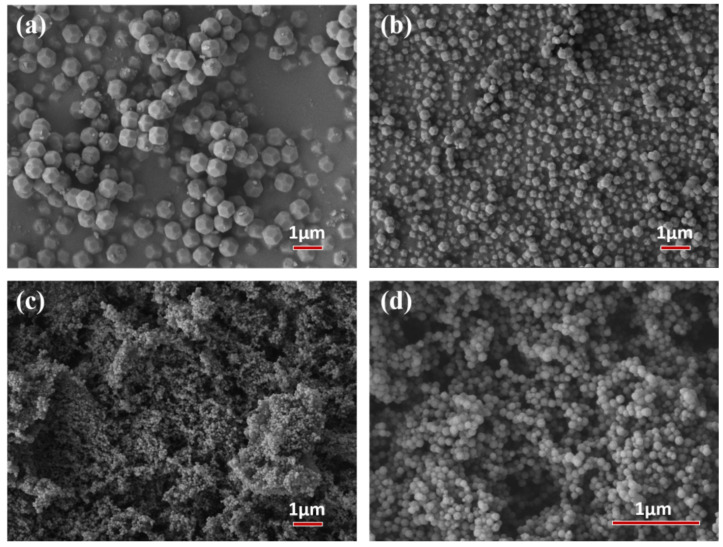
SEM images of (**a**) ZIF−8−L; (**b**) ZIF−8−M; (**c**,**d**) ZIF−8−S particles.

The nucleation rate and the crystal growth rate were two key factors in adjusting the size of the ZIF−8 particles. The particle size of ZIF−8 in this work was tuned by varying the molar ratio of the precursors, as shown in Table S1. With the molar ratio of Hmim and Zn^2+^ increasing from 2:1 to 4:1, the particle size of the ZIF−8 nanoparticles reduced sharply from ~610 nm to ~80 nm, as shown in Figure 1. This was ascribed to the fact that more Hmim would create a greater number of deprotonated nitrogen nuclei, resulting in more and smaller ZIF−8 particles. All ZIF−8 particles showed a regular rhombic dodecahedron structure and exhibited good dispersity, which might be beneficial to the membrane preparation. 

**Figure 2:**
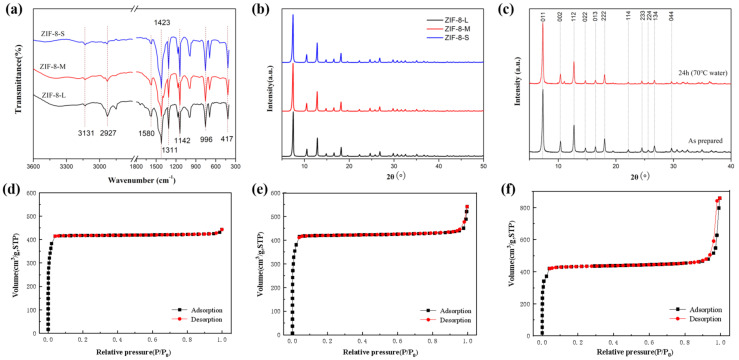
FTIR (**a**), XRD spectra (**b**,**c**), and N_2_ adsorption/desorption isotherms of ZIF−8 particles (**d**) ZIF−8(L); (**e**) ZIF−8(M); (**f**) ZIF−8(S).

The chemical and crystalline structures of ZIF−8 nanoparticles were identified by FT-IR and XRD analysis (Figure 2a–c). ZIF−8 particles with different sizes showed similar characteristic absorption peaks, which also correlated well with the literature data [34]. As shown in Figure 2a, the absorption bands at 3131 cm^−1^ and 2927 cm^−1^ were assigned to aromatic and aliphatic C-H stretching in the imidazole ring and the methyl group, respectively. The peak at 1580 cm^−1^ was ascribed to the C=N stretching in the imidazole ring. The bands at 1142 and 996 m^−1^ corresponded to C-N stretching in the imidazole ring. The peak at 417 cm^−1^ resulted from the Zn-N stretching. As shown in Figure 2b, the diffraction peaks at 2θ of 7.30°, 10.35°, 12.70°, 14.80°, 16.40°, and 18.00° corresponded to the 011, 002, 112, 022, 013, and 222 faces, respectively, which was consistent with the crystalline structure of ZIF−8 particles reported in the literature [28]. To evaluate the stability of ZIF−8 particles in the membrane preparation process, ZIF−8 particles were immersed in water at 70 °C for 24 h and then characterized by XRD, as shown in Figure 2c. It was found that ZIF−8 particles maintained high crystallinity and were clearly impervious to the water solvents at 70 °C for 24 h, exhibiting good hydrothermal stability in the preparation process. 

The N_2_ adsorption isotherms of the prepared ZIF−8 nanoparticles with different particle sizes all exhibited a general Type I adsorption isotherm and confirmed the microporous properties of the ZIF−8 particles (Figure 2d–f). The N2 adsorption experiments were conducted twice, and similar results were obtained. The BET surface area of the three types of ZIF−8 nanoparticles was 1813, 1780, and 1761 m^2^/g for ZIF−8(L), ZIF−8(M), and ZIF−8(S), respectively, indicating that all ZIF−8 particles possessed a high specific area and a perfect microporous structure. Considering the minor change in the N2 adsorption isotherm and the BET surface area, it was concluded that there was no specific correlation between ZIF−8 particle size and BET surface area [28]. As shown in Table 1 and Figure S1, the total pore volume and average pore size of the ZIF−8 particles decreased as the particle size increased, implying that the larger ZIF−8−L possessed a similar micro-pore volume, but significantly fewer meso- and macro-pores than the smaller ones. ZIF−8−S maintained the minimum micro-pore diameter with the largest average pore diameter.

#### 3.1.2. ZIF−8/PEG Mixed Matrix Membranes

**Figure 3:**
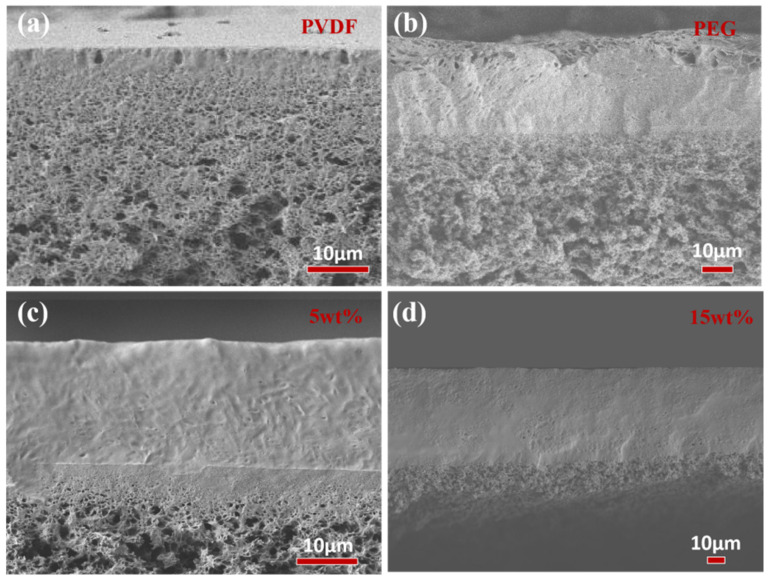
SEM images of the membrane cross-section: (**a**) PVDF substrate; (**b**) pure PEG membrane; (**c**) ZIF−8/PEG-5; (**d**) ZIF−8/PEG-15.

**Figure 4:**
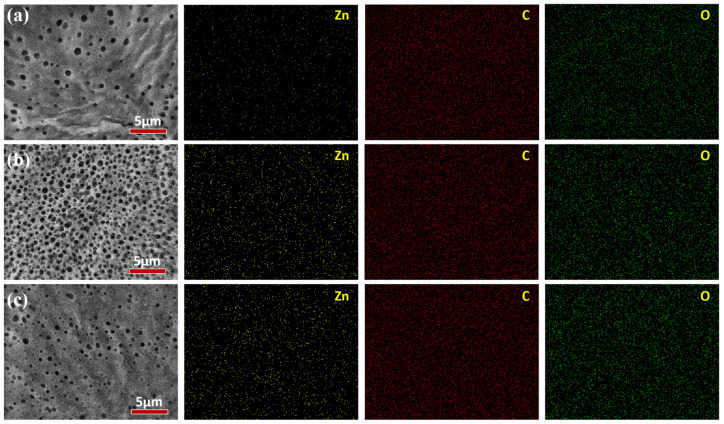
SEM images of the membrane surface (**a**) ZIF−8/PEG-5; (**b**) ZIF−8/PEG-10; (**c**) ZIF−8/PEG-15.

The morphology of ZIF−8/PEG MMMs were characterized by SEM and EDS, as shown in Figure 3 and Figure 4. As shown in Figure 3, porous PVDF exhibited sponge-like pores in the cross-section. The PEG selective layer integrated tightly with the PVDF support layer, and no obvious interface defects were observed, which might be ascribed to the leakage of PEG into the substrate. There was no obvious ZIF−8 particle aggregation in the cross-section of ZIF−8/PEG MMMs, even with 15 wt% particle loading. However, unlike common composite membranes, an abundance of blowholes appeared on the membrane surface and the cross-section near the membrane surface, which might be ascribed to the footprints left by the rapid volatilization of the solvent and the TMA catalyst molecules in the cross-linking process. Most portions of the cross-section of the selective layer were dense, especially for the ZIF−8/PEG MMMs. The porous membrane surface might be favorable to the sorption of thiophene/n−heptane molecules, and the dense portion of the PEG layer might provide enough selectivity. Based on the EDS analysis, the Zn^2+^ atom distributed homogeneously on the membrane surface, and the density of Zn^2+^ gradually increased with increasing particle loading, indicating that ZIF−8 dispersed evenly in the MMMs.

**Figure 5:**
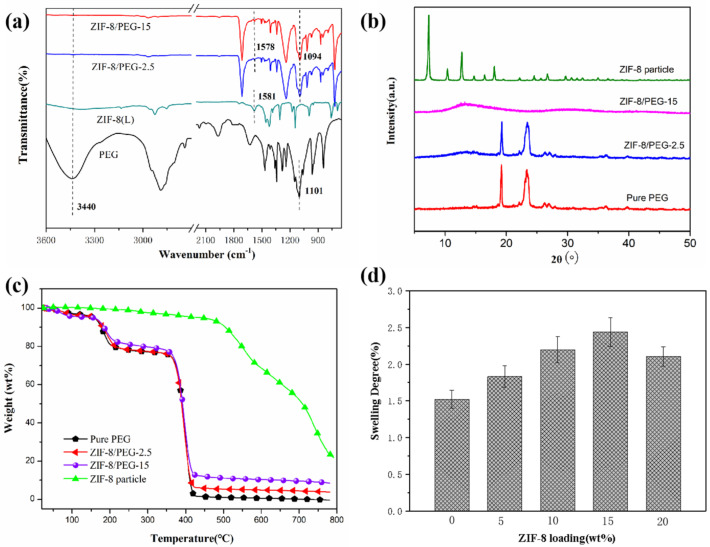
FTIR (**a**), XDR spectra (**b**), TGA (**c**) and swelling degree (**d**) of ZIF−8/PEG MMMs.

Figure 5a shows the FT-IR spectra of the ZIF−8 particles, the pure PEG membrane, and the ZIF−8/PEG MMMs. The absorption peak at 1113 cm^−1^ was assigned to the C-O-C symmetrical stretching vibration in PEG. The characteristic peak of C=N stretching in the imidazole ring at 1581 cm^−1^ of ZIF−8 also appeared in the FT-IR spectra of the ZIF−8/PEG MMMs, which suggested that ZIF−8 was successfully incorporated into the PEG membranes. Compared with the pristine PEG membrane and ZIF−8, no new characteristic absorption peaks appeared, which indicated that ZIF−8 was physically incorporated into PEG. 

XRD patterns of ZIF−8, pure PEG, and ZIF−8/PEG MMMs are shown in Figure 5b. As shown in Figure 5b, pure PEG membrane exhibited distinct and sharp diffraction peaks at 19.18°and 23.59°, which were ascribed to the crystal plane of (120) and (112). The XRD diffraction patterns of ZIF−8/PEG-2.5 MMMs indicated a crystal structure similar to that of pure PEG membrane. The position of the peaks remained intact and suggested that the crystalline structure of PEG was preserved after the incorporation of ZIF−8 particles. However, the sharp diffraction peaks of PEG were replaced by wide diffused peaks as ZIF−8 loading increased to 15 wt%, which indicated that the incorporation of ZIF−8 particles might hinder the crystallization ability of the PEG chains and result in a low degree of crystallinity. The characteristic diffraction peaks of ZIF−8 particles were not observed in the XRD diffraction patterns of ZIF−8/PEG MMMs, which might be due to the coverage of the PEG molecules and the detection depth of XRD.

The thermal property parameters of the polymer of PEG and MMMs, including the melting and crystallization temperature, are recorded in Table 2. The DSC curves of pure PEG membrane and ZIF−8/PEG MMMs are shown in Figure S4. As shown in Table 2, ZIF−8/PEG MMMs exhibited a higher melting and crystallization temperature (Tm and Tc) than pure PEG membrane, as well as lower melting latent heats. The increased Tm and Tc resulted from the reduced chain mobility within the polymer matrix, and the lower melting latent heats revealed the lower crystallinity of the MMMs. A similar trend could be found in other MMMs. The reduced chain mobility might be ascribed to the denser chain packing in the vicinity of the interface due to the strong interaction between the two phases and the chain rigidification effect [35]. The depressed chain mobility of PEG might also have hindered the regular chain packing and contributed to the lower crystallinity of PEG (as shown in Table 2).

The thermal stability of the ZIF−8 particles, pure PEG membrane, and ZIF−8/PEG MMMs were investigated by TGA, as shown in Figure 5c. The thermal decomposition temperature of the ZIF−8 particles was over 500 °C, which showed high thermal stability. The slight weight loss of PEG membranes below 100 °C resulted from the evaporation of water molecules adsorbed into the membranes. The weight loss between 150 °C and 200 °C might be ascribed to the evaporation of excessive citric acid with a sublimation temperature of 175 °C, as the cross-linking reagent was overdosed in the preparation process. Compared with that of pure PEG membrane, it was shown that the thermal stability of ZIF−8/PDMS MMMs was strongly improved with an increased degradation temperature and a higher residue mass.

To evaluate the swelling resistance of the membranes, the degrees of swelling of pure PEG membrane and ZIF−8 /PEG MMMs in 500 ppm thiophene/hexane solution were measured, as shown in Figure 5d. The swelling degree of all ZIF−8/PEG MMMs was below 3%, exhibiting sufficient swelling resistance. It was found that the swelling degree increased as ZIF−8 loading increased from 0 wt% to 15 wt%, and then decreased with increasing ZIF−8 loading furthermore. There were several factors influencing the swelling properties of MMMs: (1) the porous ZIF−8 particles could hold more small molecules; (2) the decreased crystallinity of PEG due to the incorporation of ZIF−8 particles might favor the adsorption of small molecules; and (3) the chain rigidification due to denser chain packing in the vicinity of the interface might decrease the swelling degree. Several factors competed together and resulted in the variation of the swelling ratio with the augmentation of ZIF−8 loading. 

### 3.2. Molecular Simulation

**Figure 6:**
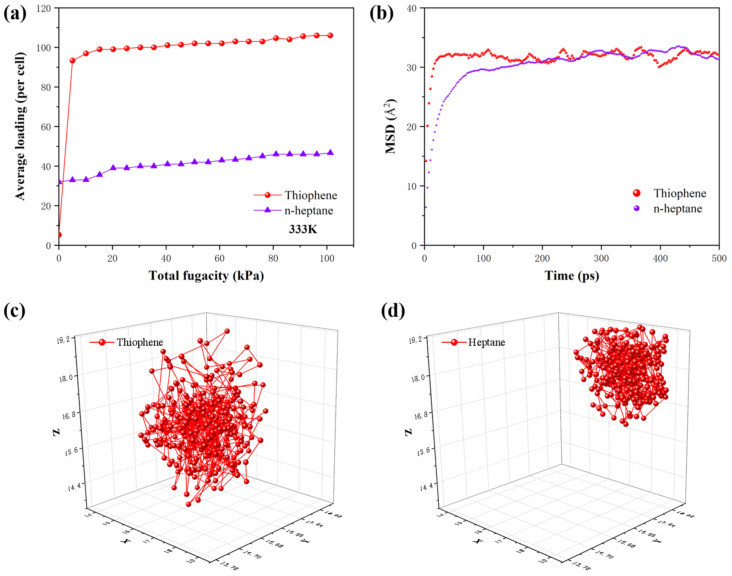
Adsorption isotherm (**a**), MSD (**b**), diffusion trajectory of thiophene (**c**), and n−heptane (**d**) molecules in ZIF−8 at 333 K.

Figure 6 showed the adsorption isotherms (a) and the MSD (b) of the thiophene and n−heptane molecules in ZIF−8 at 333 K. As shown in Figure 6a, the adsorption capacity of ZIF−8 for thiophene was much higher than that for n−heptane, which indicated that ZIF−8 exhibited a preferential adsorption for thiophene molecules. The incorporation of ZIF−8 particles into PEG might enhance the sorption selectivity of membranes. The dynamic simulation tests were conducted three times, and similar results were obtained. As shown in Figure 6b, the slope of MSD for thiophene was much larger than that for n−heptane, which indicated that the diffusion coefficient of thiophene might be larger than that of thiophene in ZIF−8 [36]. The trajectory of the thiophene and n−heptane molecules also confirmed this conclusion, as shown in Figure 6c,d. The diffusion trajectory range of thiophene was significantly larger than that of n−heptane, which also suggested the higher diffusion rate of the thiophene molecules. The simulation results indicated that ZIF−8 particles possessed both high sorption and diffusion selectivity for thiophene, which might be favorable to enhancing membrane selectivity and permeability. 

### 3.3. Pervaporation Performance of the Membranes

#### 3.3.1. Effect of ZIF−8 Particle Size

**Figure 7:**
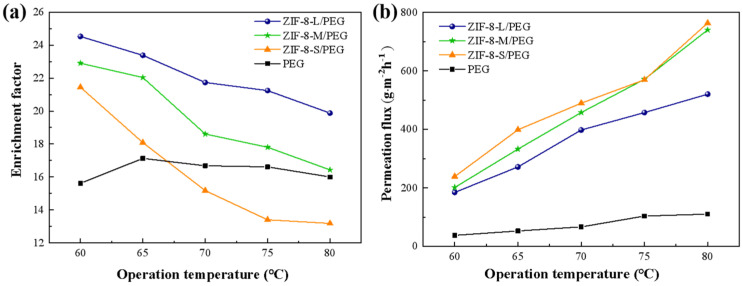
Effect of ZIF−8 particle size on pervaporation performance of the ZIF−8/PEG MMMs (**a**) enrichment factor and (**b**) permeation flux.

**Figure 8:**
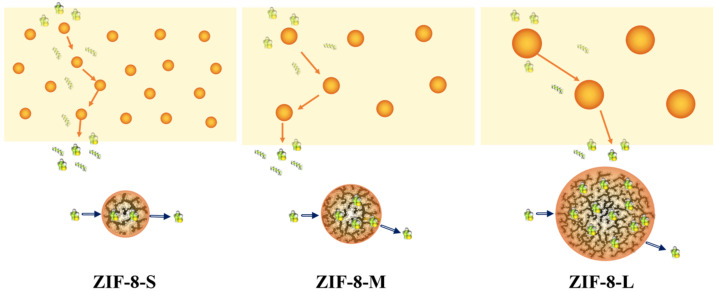
Schematic diagram of transport mechanisms occurring in ZIF−8/PEG MMMs with different particle sizes.

The effect of ZIF−8 particle size on the pervaporation performance of the ZIF−8/PEG MMMs (ZIF−8 loading of 15 wt%) in the range of 60–80 °C is shown in Figure 7. It was found that PEG-based MMMs filled with larger ZIF−8 particles showed much higher sulfur enrichment factor and lower permeation flux than those with smaller ZIF−8 particles. ZIF−8−L/PEG MMMs reached a maximum sulfur enrichment factor of 24.5 with a permeation flux of 183.2 g·m^−2^·h^−1^ at 60 °C, which was much higher than those of pure PEG and ZIF−8−M/PEG MMMs, as well as ZIF−8−S/PEG MMMs. This was probably due to the intrinsic differences in the porous structure of ZIF−8 and the connectivity of the transport channels created by the ZIF−8 particles. The transport mechanism in PEG MMMs made up of different ZIF−8 particle size is proposed in Figure 8. Larger ZIF−8−L particles provided more and longer selective transport channels in one single particle compared with smaller ZIF−8−S particles, which might contribute to the higher selectivity for thiophene but lower diffusion rate. Smaller ZIF−8−S possessed similar micro-pore volumes, but significantly more meso- and macro-pores than larger ones, which might provide more larger porous channels for small molecules and contribute to higher permeation flux while sacrificing some selectivity. Moreover, the number of ZIF−8−L particles in the MMMs was lower than that of the smaller particles with same particle loading, which might weaken the connectivity between adjacent ZIF−8−L nanoparticles and result in low-efficiency molecular transport in the membrane. The distance among ZIF−8−S particles in MMMs was smaller than that of ZIF−8−L with the same particle loading, which might provide relatively continuous transport channels and be beneficial to rapid diffusion across membrane. Besides, the surface area available for the mass transport was larger for MMMs with ZIF−8−S particles due to the larger specific surface area of the smaller ZIF−8 particles, which might also contribute to higher permeability in ideal MMMs. Monsalve-Bravo et al. [37] also reported similar results showing the increasing permeability of MMMs resulting from decreasing the particle size. 

#### 3.3.2. Effect of ZIF−8 Loading

**Figure 9:**
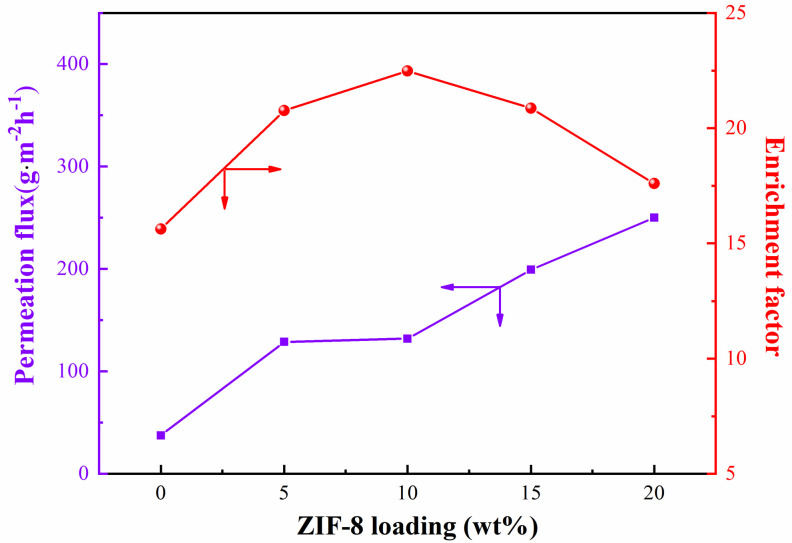
The effect of ZIF−8 loading on the pervaporation performance of ZIF−8−L/PEG MMMs.

The effect of ZIF−8−S loading on the pervaporation performance of ZIF−8−S/PEG MMMs was investigated, as shown in Figure 9. With increasing ZIF−8 loading from 0 wt% to 20 wt%, the total flux continuously increased, while the sulfur enrichment factor increased at first and then decreased. The sulfur enrichment factor reached the maximum of 22.5 with ZIF−8 loading of 10 wt%. Compared with pure PEG membrane, the enhancement of both the sulfur enrichment factor and the total flux of ZIF−8/PEG MMMs might be ascribed to several reasons. First, porous ZIF−8 particles provided a larger thiophene adsorption capacity than that of n−heptane, as confirmed by molecular simulation, which might be attributed to the adsorption sites provided by ZIF−8 based on Zn^2+^ and S coordination interaction [22]. Moreover, the rich porous structure and large specific surface area of ZIF−8 contributed fast and thiophene-selective transport channels. Moreover, the good integration between ZIF−8 particles and PEG matrix might have created a more selective interface. Last, the decreased PEG crystallinity with increasing ZIF−8 loading might increase the free volume of PEG polymers, which was also favorable to the diffusion of thiophene with a larger dynamic diameter. However, as ZIF−8 loading exceeded 10 wt%, the sulfur enrichment factor decreased, and the permeate flux increased rapidly. This might have resulted from the particle agglomeration due to excessive ZIF−8 loading, which might create non-selective voids among particle aggregates and destroy the connectivity of the transport channels provided by the ZIF−8 particles.

#### 3.3.3. Effect of Operation Temperature

Figure 7 also showed the effect of operation temperature on the pervaporation desulfurization performance of ZIF−8/PEG MMMs. As the temperature increased from 60 °C to 80 °C, the sulfur enrichment factor decreased and permeation flux increased continuously. This might have resulted from the increased driving forces and diffusion coefficient of the small molecules with increasing operation temperature. Moreover, the swelling of the ZIF−8/PEG MMMs might be enhanced by increasing temperature, which also enhanced the diffusion of small molecules. The permeation activation energy is calculated according to the Arrhenius equation, as listed in Table 3. It can be seen that the apparent Ep of thiophene for all the ZIF−8/PEG MMMs was smaller than that of n−heptane, while PEG showed the opposite tendency, which indicated that the permeation of thiophene across the ZIF−8/PEG MMMs was simpler than for n−heptane. The apparent Ep of n−heptane for all the ZIF−8/PEG MMMs was larger than that of thiophene, which suggested that n−heptane permeation was more sensitive to operation temperature than was thiophene. Therefore, the increase in n−heptane permeation flux was more significant than that of thiophene, resulting in the decrease in the sulfur enrichment factor with increasing temperature.

#### 3.3.4. Effect of Feed Concentration

**Figure 10:**
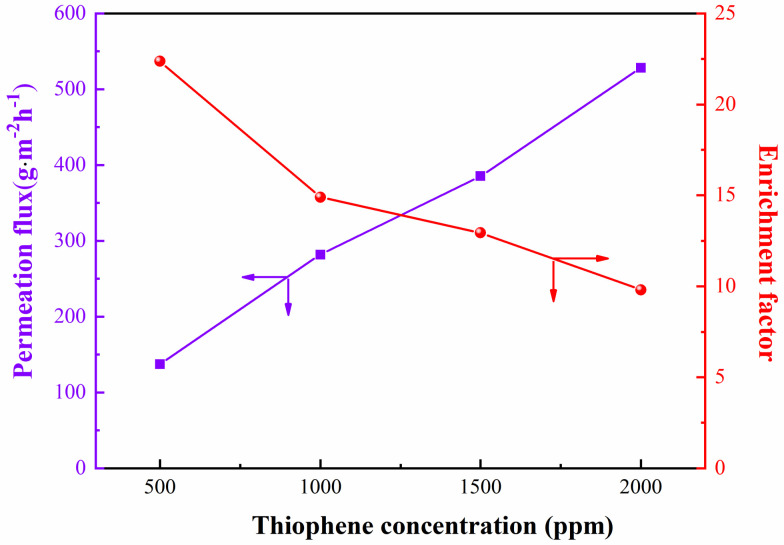
Effect of feed concentration on the pervaporation performance of thiophene (**a**) and n−heptane permeation flux of (**b**) on the ZIF−8/PEG MMMs.

The effect of feed concentration on the pervaporation performance of ZIF−8−L/PEG MMMs was investigated, as shown in Figure 10. With increasing feed concentration, the total flux increased, while the sulfur enrichment factor decreased. This might be attributed to the ZIF−8 particles have a strong affinity for thiophene. As the thiophene concentration in the feed increased from 500 ppm to 2000 ppm, the membrane swelling might be enhanced due to the strong adsorption capacity of ZIF−8 particles for thiophene. As the dynamic diameter of n−heptane (0.43 nm) was smaller than that of thiophene (0.53 nm), the increase in n−heptane permeate flux due to membrane swelling might be much higher than that of thiophene, resulting in a decrease in the sulfur enrichment factor.

#### 3.3.5. Comparison of Desulfurization Performances

**Figure 11:**
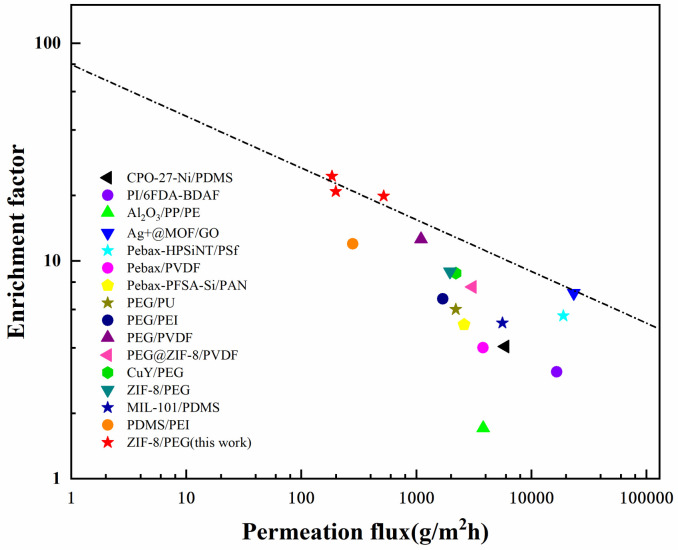
Desulfurization performance comparison of ZIF−8/PEG MMMs with other membranes.

Compared with other desulfurization membranes, ZIF−8/PEG MMMs exhibited a higher enrichment factor, but much lower permeation flux, as shown in Figure 11. The enhancement of the enrichment factor was mainly attributed to the selective transport channels and adsorption sites provided by ZIF−8 based on Zn^2+^ and S coordination interaction, which facilitated the selective sorption and mass transfer of the thiophene molecules. The much lower permeation flux might have resulted from the higher molecular weight of the PEG chains and the greater thickness of the selective layer, as well as the lower operation temperature. Moreover, the intrusion of PEG polymers into the PVDF porous substrate also increased the mass transport resistance, which decreased the permeation flux. Future work should concentrate on the improvement of the permeation flux of ZIF−8/PEG MMMs without sacrificing the enrichment factor by decreasing the membrane thickness of the selective layer and pre-wetting the substrate to prevent polymer chain intrusion. The interfacial micro-structure and intrinsic sorption–diffusion properties of guest molecules in the ZIF−8 particles might play an important role in desulfurization performance, which should be investigated further to clarify the mass transfer mechanism of guest molecules in MMMs.

## 4. Conclusions

ZIF−8/PEG MMMs were prepared by incorporating ZIF−8 with different particle sizes into the PEG matrix. Based on FT-IR, XRD, SEM, and BET analysis, it was found that the three kinds of ZIF−8 prepared in this work all possessed a specific crystalline structure, with high specific area and microporous volumes. The total pore volume and average pore size of ZIF−8 particles decreased as the particle size increased, implying that the larger ZIF−8−L particles possessed similar micro-pore volumes, but significantly fewer meso- and macro-pores than smaller ZIF−8 particles. ZIF−8−S maintained the minimum micro-pore diameter with the largest average pore diameter. ZIF−8 particles distributed homogeneously on the membrane surface and in the cross-section of the ZIF−8/PEG MMMs. The incorporation of ZIF−8 into the PEG matrix decreased the crystallinity and enhanced the swelling degree of the PEG polymers. PEG-based MMMs made up of larger ZIF−8−L particles showed a much higher sulfur enrichment factor and a slightly lower permeation flux than those with smaller ZIF−8 particles. ZIF−8−L/PEG MMMs reached a maximum sulfur enrichment factor of 24. 5 with a permeation flux of 183.2 g·m^−2^·h^−1^ at 60 °C, which was much higher than that of pure PEG, ZIF−8−M/PEG MMMs, or ZIF−8−S/PEG MMMs. This was probably due to the intrinsic differences in the porous structure of ZIF−8 and the connectivity of the transport channels created by the ZIF−8 particles. Larger ZIF−8−L provided more and longer selective transport channels in one single particle compared with smaller ZIF−8−S, which might contribute to a higher selectivity for thiophene, but a lower diffusion rate. Moreover, the number of ZIF−8−L particles in MMMs was lower than that of smaller particles with same particle loading, which might weaken the connectivity between adjacent ZIF−8−L nanoparticles and result in low-efficiency molecular transport in the membrane. Moreover, the surface area available for the mass transport was smaller for MMMs with ZIF−8−L particles due to the smaller specific surface area of the ZIF−8−L particles, which might have also resulted in lower permeability in the ZIF−8−L/PEG MMMs. With increasing ZIF−8 loading from 0 wt% to 20 wt%, the total flux increased continuously, while the sulfur enrichment factor increased at first and then decreased. This work might provide some new insights into the investigation of the particle size effect on the pervaporation performance of MMMs. 

## Figures and Tables

**Table 1:** Physical properties of ZIF−8 nanoparticles of different particle size.

	Particle Diameter (nm)	BET Surface Area (m^2^/g)	Micropore Diameter (nm)	Average Pore Size (nm)	Pore Volume (cm^3^/g)		
					Total	<2.0 nm	>2.0 nm
ZIF−8−L	~610	1813	0.638	1.510	0.685	0.640	0.045
ZIF−8−M	~270	1780	0.643	1.883	0.838	0.639	0.199
ZIF−8−S	~80	1761	0.633	3.018	1.328	0.642	0.686

**Table 2:** Melting and crystallization properties of pure PEG membrane and ZIF−8/PEG MMMs.

*Sample*	*PEG Mass Fraction*	*T_m_ (°C)*	*T_c_ (°C)*	∆*H_m_ (J/g)*	*Crystallinity* *(%)*
pure PEG membrane	1.00	59.80	33.99	106.5	52.0
ZIF−8−L/PEG-5	0.95	60.84	36.86	104.9	51.2
ZIF−8−L/PEG-10	0.90	61.13	35.55	99.46	48.5
ZIF−8−L/PEG-15	0.85	63.56	37.11	87.71	42.8

**Table 3:** Permeation activation energy of thiophene and heptane in various ZIF−8/PEG MMMs.

*Molecules*	*Permeation Activation Energy (kJ/mol)*			
	*PEG*	*ZIF−8−L/PEG*	*ZIF−8−M/PEG*	*ZIF−8−S/PEG*
Thiophene	56.0	38.0	44.5	33.2
n−heptane	55.6	51.1	61.9	52.8

## Data Availability

Not applicable.

## Supplementary Materials

The following supporting information can be downloaded at: https://www.mdpi.com/article/10.3390/membranes13050515/s1, Figure S1: Schematic diagram of the preparation process of ZIF−8/PEG MMMs; Figure S2: Schematic diagram of the pervaporation apparatus; Figure S3: Pore diameter distribution of ZIF−8 particles via HK (up) and DFT (down) analysis (a,d) ZIF−8−L; (b,e) ZIF−8−M; (c,f) ZIF−8−S; Figure S4: DSC spectra of ZIF−8/PEG MMMs (a) crystallization spectra; (b) melting spectra. Table S1: Synthesis conditions for ZIF−8 nanoparticles of different particle size.
