# Supplementary material for: Enhanced Desulfurization Performance of ZIF−8/PEG MMMs: Effect of ZIF−8 Particle Size
Source: Membranes (Basel). 2023 May 15;13(5):515. doi: 10.3390/membranes13050515 (PMC10224113; doi:10.3390/membranes13050515)
Supplement: Supplementary file 1 [file membranes-13-00515-s001.zip › membranes-2392434-supplementary.pdf]

Enhanced desulfurization performance of ZIF-8/PEG MMMs: Effect of ZIF-8 particle size

Xia Zhan[a,b*], Kaixiang Gao[a], Yucheng Jia[a], Ning Liu[a], Wen Deng[a], Xuebin Guo[a], Hehe Li[a], Jiding Li[c]

(a. China Food Flavor and Nutrition Health Innovation Center, Beijing Technology and Business University, Beijing 100048, China; b. Key Laboratory of Cleaner Production and Integrated Resource Utilization of China National Light Industry, Beijing Technology and Business University, Beijing 100048, China; c. The State Key Laboratory of Chemical Engineering, Department of Chemical Engineering, Tsinghua University, Beijing 100084, PR China)

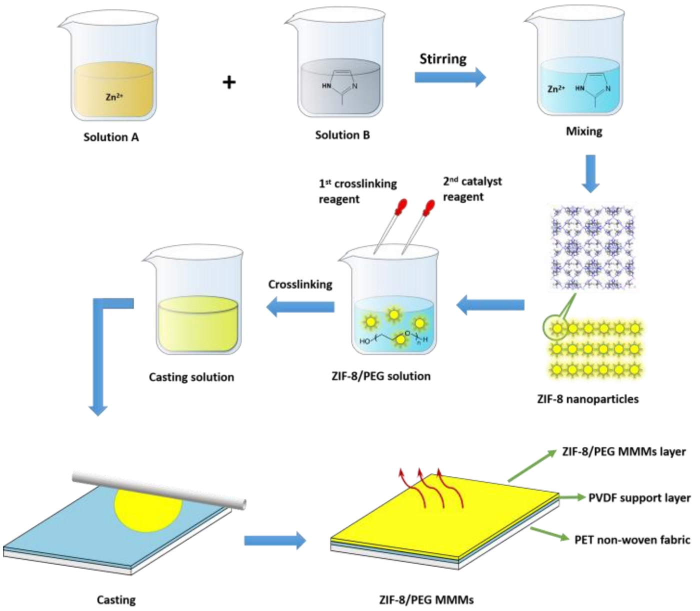

Fig.S1 Scheme diagram of preparation process of ZIF-8/PEG MMMs

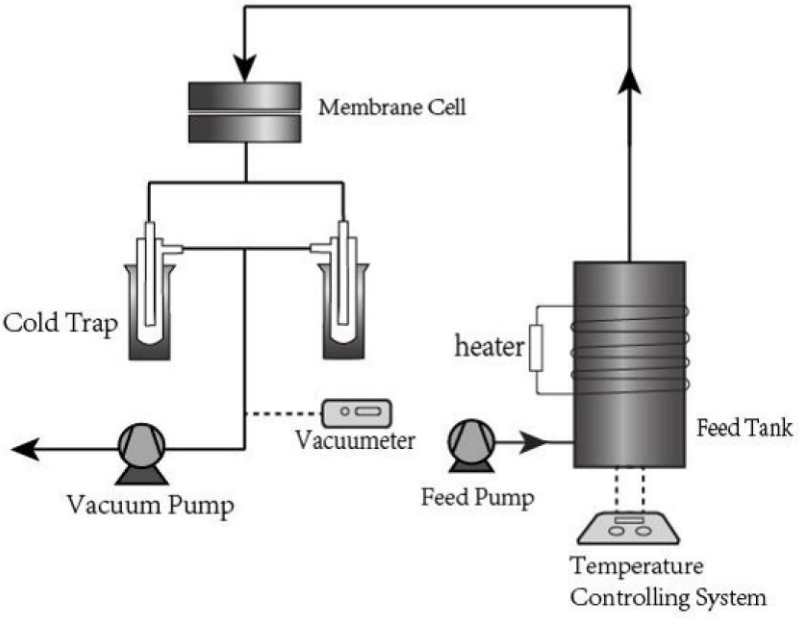

Fig.S2 Scheme diagram of pervaporation apparatus

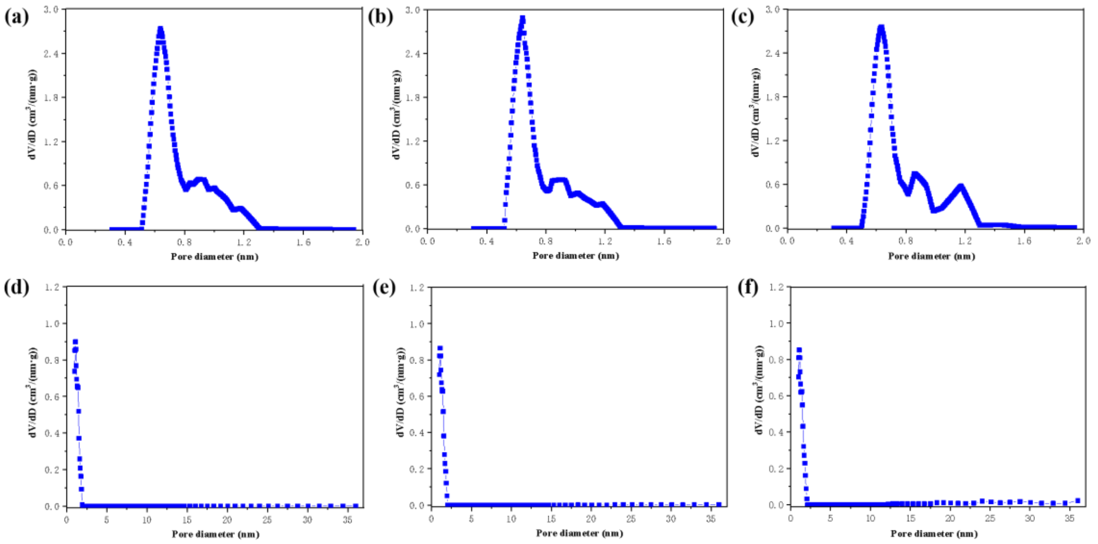

Fig.S3 Pore diameter distribution of ZIF-8 particles via HK(up) and DFT(down) analysis (a,d)ZIF-8-L; (b,e)ZIF-8-M; (c,f)ZIF-8-S

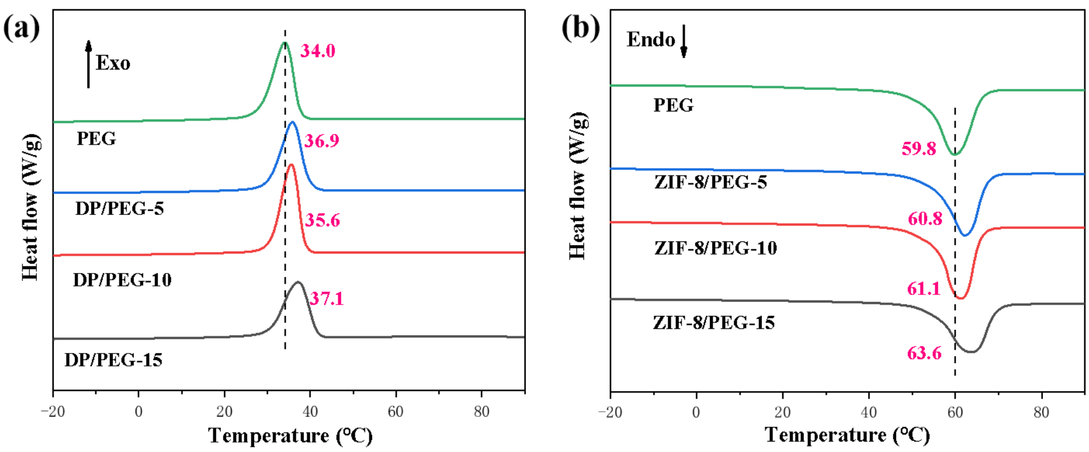

Fig.S4 DSC spectra of ZIF-8/PEG MMMs(a) Crystallization spectra (b) Melting spectra

Table S1 Synthesis conditions for ZIF-8 nanoparticle of different particle size

	ZIF-8(L)	ZIF-8(M)	ZIF-8(S)
Zn^{2+} concentration(mmol)	0.01	0.01	0.01
Hmim concentration(mmol)	0.02	0.03	0.04
Solvent	Methanol	Methanol	Methanol
Reaction time(h)	24	24	24
Particle size(nm)	~610	~270	~80
